# Evolution determines how global warming and pesticide exposure will shape predator–prey interactions with vector mosquitoes

**DOI:** 10.1111/eva.12390

**Published:** 2016-06-07

**Authors:** Tam T. Tran, Lizanne Janssens, Khuong V. Dinh, Lin Op de Beeck, Robby Stoks

**Affiliations:** ^1^ Institute of AquacultureNha Trang UniversityNha TrangVietnam; ^2^ Laboratory of Aquatic Ecology, Evolution and ConservationUniversity of LeuvenLeuvenBelgium; ^3^ National Institute of Aquatic ResourcesTechnical University of DenmarkCopenhagenDenmark

**Keywords:** biological control, climate change, contaminants, *Ischnura elegans*, latitudinal gradient, life history evolution, range shifts, thermal evolution

## Abstract

How evolution may mitigate the effects of global warming and pesticide exposure on predator–prey interactions is directly relevant for vector control. Using a space‐for‐time substitution approach, we addressed how 4°C warming and exposure to the pesticide endosulfan shape the predation on *Culex pipiens* mosquitoes by damselfly predators from replicated low‐ and high‐latitude populations. Although warming was only lethal for the mosquitoes, it reduced predation rates on these prey. Possibly, under warming escape speeds of the mosquitoes increased more than the attack efficiency of the predators. Endosulfan imposed mortality and induced behavioral changes (including increased filtering and thrashing and a positional shift away from the bottom) in mosquito larvae. Although the pesticide was only lethal for the mosquitoes, it reduced predation rates by the low‐latitude predators. This can be explained by the combination of the evolution of a faster life history and associated higher vulnerabilities to the pesticide (in terms of growth rate and lowered foraging activity) in the low‐latitude predators and pesticide‐induced survival selection in the mosquitoes. Our results suggest that predation rates on mosquitoes at the high latitude will be reduced under warming unless predators evolve toward the current low‐latitude phenotype or low‐latitude predators move poleward.

## Introduction

How global warming will affect vector species and associated diseases is one of the pressing questions with relevance for human health (Kovats et al. 2001; Ramasamy and Surendran 2012; Parham et al. 2015). While much attention is going to how vectorborne disease dynamics will change in a warmer world, much less attention is going to how warming will shape biotic interactions with vector species (Parham et al. 2015). Yet, biotic interactions such as predator–prey interactions may be an important factor controlling vector mosquitoes (Kamareddine 2012). Despite the general insight that predator–prey interactions are important for the local persistence of prey populations under global warming (Gilman et al. 2010; Zarnetske et al. 2012), few studies directly looked at how warming affects the outcome of these interactions (but see, e.g., De Block et al. 2013; Hayden et al. 2015). Moreover, none of these studies considered vector prey species. Another challenge for understanding how predators may control vector populations is that in many areas, pest control provided by natural enemies has been lowered by the use of pesticides (MEA 2005). Moreover, pesticide use is expected to increase under global warming (Kattwinkel et al. 2011). Therefore, to assess the future potential of predation to play a role in vector control in a warming world, we need to study how predator–prey interactions are jointly shaped by warming and pesticides (Schmitz and Barton 2014).

Many species have the potential to evolve in response to warming (Merilä and Hendry 2014; Stoks et al. 2014). Therefore, a relevant applied question in this context is whether gradual thermal evolution of the predator may mitigate how warming and pesticide exposure shape predator–prey interactions with vector species, and with pest species in general (Roderick et al. 2012). Importantly, gradual evolution under global warming may thereby also shape the vulnerability to pesticides. Indeed, adaptation to a warmer climate may come at the cost of a reduced tolerance to contaminants (Moe et al. 2013). Besides direct effects of thermal evolution, also indirect effects mediated through evolved changes in life history, particularly in voltinism (number of generations per year), may affect the vulnerability to pesticides (e.g., Dinh Van et al. 2014a). Indeed, at warmer temperatures, invertebrates typically show more generations per year and in accordance evolve a faster growth and development as each generation will have less time to complete the larval stage (Seiter and Kingsolver 2013). Based on life history theory, a faster life history will come at the cost of a reduced investment in other functions, including detoxification and repair (Sibly and Calow 1989; Congdon et al. 2001).

A powerful way to assess the potential of gradual thermal evolution (being direct or indirect) in shaping trait evolution is to study besides high‐latitude populations at their current temperature and the predicted higher temperature under warming, also low‐latitude populations currently living at the higher temperature predicted at the high latitude under global warming. Such space‐for‐time substitution approach (Fukami and Wardle 2005; De Frenne et al. 2013) has only been rarely applied in the context of predator–prey interactions (but see De Block et al. 2013) and ecotoxicology (but see Janssens et al. 2014). Instead, the few studies on warming effects on predator–prey interactions typically applied a ‘step‐increase’ temperature experiment at one latitude (e.g., Rall et al. 2010; Miller et al. 2014; Hayden et al. 2015; Sentis et al. 2015). Such studies, however, do not allow assessing the role of long‐term gradual evolution in mediating the impact of a temperature increase and the associated changes in sensitivity to contaminants.

To better understand how warming and pesticides will shape the outcome of predator–prey interactions, it is important to expose both predator and prey to these stressors. Yet, the few studies that manipulated both stressors only exposed the prey (e.g., Broomhall 2002, 2004) or the predators (Dinh Van et al. 2014a). More general, most studies on the effect of pesticides on predator–prey interactions only exposed the predators (e.g., Dinh Van et al. 2014b) or the prey (e.g., Brooks et al. 2009; Reynaldi et al. 2011). Yet, joint exposure of both predator and prey, the likely field scenario, may have strongly different outcomes (Junges et al. 2010; Englert et al. 2012; Rasmussen et al. 2013). Moreover, the relatively few studies that exposed both predator and prey to a pesticide, mostly scored the behavior of only one interactor, thereby precluding a full understanding of how pesticides change the outcome of predator–prey interactions (Schulz and Dabrowski 2001; Rasmussen et al. 2013).

In the current study, we tested how warming and exposure to a pesticide shape predator–prey interactions in the larval stage between a vector mosquito and important invertebrate predators, damselfly larvae. We explicitly considered the potential of thermal evolution of the predator in high‐latitude populations in modifying these effects by applying a space‐for‐time substitution approach where we studied triplicated low‐ and high‐latitude populations of the damselfly predators. Moreover, to get a mechanistic understanding of how both stressors change the outcome of predator–prey interactions, we studied the behavior of both antagonists when they were exposed to the stressors in a factorial way. Damselfly larvae are important natural predators of mosquitoes (Klecka and Boukal 2012) and are used as biological control agent (e.g., Mandal et al. 2008). The predator was the damselfly *Ischnura elegans* (Vander Linden, 1820), whose latitudinal differentiation in life history is well characterized (e.g., Shama et al. 2011; Stoks et al. 2012). The prey species was *Culex pipiens* (Linaeus, 1758) form molestus, a member of the *C. pipiens* complex, which is an important vector of pathogens such as West Nile virus and St. Louis encephalitis virus (Becker et al. 2010; Farajollahi et al. 2011). We chose the pesticide endosulfan, an organochlorine insecticide, that has been widely used to control vector mosquitoes (Calamari and Naeve 1994). This pesticide has been reported to increase the vulnerability of aquatic invertebrates to predation (e.g., Janssens and Stoks 2012; Trekels et al. 2013).

## Materials and methods

### Experimental design

We investigated the combined impact of warming and pesticide exposure on predator–prey interactions between damselflies and mosquitoes using a full factorial design with two predator latitudes (low‐ versus high‐latitude damselflies) × two temperature treatments (20°C vs 24°C) × two pesticide treatments (endosulfan absent versus present). To keep the experiment feasible, we did not study mosquito populations from different latitudes; all mosquitoes came from a temperature regime matching that of the high‐latitude populations of the damselfly predators. This way we only tested for the effects of thermal evolution of the predators in high‐latitude populations.

The chosen temperatures reflect the mean summer water temperatures in shallow ponds occupied by the damselfly *Ischnura elegans* in southern Sweden (20°C) and southern France (24°C) (De Block et al. 2013). Based on simulations using the model Flake (e.g., Kirillin et al. 2011; Dinh Van et al. 2014a), the mean summer water temperature of ponds where the mosquito culture originates is about 20°C (for details see Appendix S1). Note that high‐latitude damselfly populations and the studied mosquito populations currently encounter daily summer water temperatures of 24°C, although this occurs infrequently. Indeed, based on the Flake model (Kirillin et al. 2011), the percentage of daily water temperatures during summer equal to or exceeding 24°C is ca. 3% in high‐latitude damselfly populations and 11–19% in the mosquito populations. Importantly, the 4°C difference corresponds with the predicted temperature increase by 2100 according to IPCC (2013) scenario RPC 8.5. This allows a space‐for‐time substitution where the potential impact of gradual thermal evolution in the high‐latitude predator populations can be evaluated. The comparison of the phenotypes of the high‐latitude predators at 20°C and 24°C indicates the potential of the currently present thermal plasticity (without change in the genetic constitution, hence without thermal evolution) to deal with 4°C warming. The comparison of the high‐latitude predators and the low‐latitude predators at 24°C reflects the potential of gradual thermal evolution in response to 4°C warming to shift the phenotypes of the high‐latitude populations. In addition, we also tested the low‐latitude populations at 20°C, to obtain a full factorial design where populations from both latitudes are tested at their ‘local’ mean temperature and the mean temperature of the other latitude provides a powerful design to test for local thermal adaptation (Kawecki and Ebert 2004). Both predators and prey were reared at one of the two temperatures from the egg stage onwards and afterward tested only at their rearing temperature. This way we allowed developmental, long‐term acclimatization to the experimental temperatures and avoided any abrupt thermal changes before exposing the animals to the pesticide and testing them in the predation trials. This mimics a more realistic scenario compared to testing animals directly after exposing them to a higher temperature (Seebacher et al. 2015).

The study consisted of two coupled experiments that both tested for single and combined effects of temperature increase and pesticide exposure. In the first experiment, the exposure experiment, we examined effects on survival and growth rate of predators and prey kept in isolation. In the second follow‐up experiment, the predation experiment, we studied the survival of the mosquito larvae in the presence of a lethal damselfly predator and monitored the behaviors of both predators and prey. All predator and prey individuals were kept at the same temperature‐by‐pesticide treatment during both experiments.

### Study animals and rearing

The laboratory culture of *Culex pipiens* was initiated from the stock culture housed at the Helmholtz Centre for Environmental Research – UFZ, Germany. To start up the experiments, freshly hatched mosquito larvae were reared at 20°C or 24°C until they reached the final instar (L4) (for details see Appendix S2) after which they entered the exposure experiment. A rearing temperature of 24°C was provided by placing trays in temperature‐controlled water baths in the same room.

We collected *Ischnura elegans* damselflies at two latitudes representing low‐latitude (southern France) and high‐latitude (southern Sweden and Denmark) regions of the species’ distribution range in Europe (Gosden et al. 2011). At each latitude, three populations were randomly collected, namely Saint‐Martin‐de‐Crau (43°38′16.57″N, 4°50′49.06″E), Camaret‐sur‐Aigues (44°9′1.47″N, 4°51′20.37″E) and Domaine de Valcros (43°10′9.02″N, 6°16′11.36″E) in southern France; Nöbbelövs mosse (55°44′5.98″N, 13°9′10.02″E) and Erikso (58°56′4.90″N, 17°39′21.50″E) in southern Sweden and Ahl Hage (56°10′59.64″N, 10°39′1.69″E) in Denmark. All collecting sites were shallow ponds with abundant aquatic vegetation. Except for the one French population Camaret‐sur‐Aigues, the collecting sites were not embedded by cropland and close to forest (Appendix S3) making it unlikely that they were affected by agriculture (Declerck et al. 2006). Further, damselfly larvae from Camaret‐sur‐Aigues did not differ in their response to the pesticide compared to the other two French populations (Appendix S3). Moreover, any local adaptation to pesticides would be unlikely in damselflies given the high levels of gene flow (Johansson et al. 2013).

In each damselfly population, eggs of eight mated females were collected and transferred to the laboratory in Belgium. Ten days after hatching, larvae were placed individually in 200‐mL plastic cups filled with aerated tap water. Larvae were daily fed *ad libitum* with *Artemia* nauplii (mean ± SE: 305 ± 34 nauplii per food portion, *n* = 10 food portions) 6 days a week until they reached the final instar after which they entered the exposure experiment. During the exposure period, the larvae were daily fed the same amount of *Artemia* nauplii as during the pre‐exposure period.

### Pesticide concentration

We selected an endosulfan concentration of 28 *μ*g/L based on a range finding experiment (for details see Appendix S4). In Europe, endosulfan concentrations up to 100 *μ*g/L have been detected in surface waters (Brunelli et al. 2009). We daily prepared the endosulfan exposure solution based on a stock solution of 500 *μ*g/mL dissolved in acetone (stored in the dark at 4°C). In the control treatment, we used aerated tap water instead of a solvent control, as the range finding experiment showed no significant difference in survival and growth between the water control and solvent control and this both in the mosquito larvae and in the damselfly larvae (for details see Appendix S4).

### Exposure experiment

At the start of the exposure experiment, 25 freshly molted L4 mosquito larvae of the same rearing temperature were placed in 200‐mL cups containing 125 mL control or pesticide medium. During the 5‐day exposure period, mosquito larvae were reared under the same conditions as during the pre‐exposure period. Damselfly larvae were exposed individually in the same type of cups as the mosquitoes and were daily fed the same amount of *Artemia* nauplii as during the pre‐exposure period. The medium was renewed every other day for both species. For mosquito larvae, we used 25 replicates (sets of 25 larvae, total of 625 larvae) per temperature‐by‐pesticide treatment combination. For damselfly larvae, the number of replicates varied from 8 to 15 per latitude‐by‐temperature‐by‐pesticide treatment combination (total of 97 damselfly larvae); exact sample sizes are shown in the figures.

We daily checked mortality of the two study species and adjusted the food provided to each cup with mosquitoes based on the number of living larvae in the cup. We additionally quantified growth rate based on the increase in wet mass over the exposure period for the two study species. For mosquito larvae, we obtained an estimate of the initial mean wet mass per larva based on the fresh mass of 10 randomly selected larvae entering L4 at each temperature. These larvae were carefully blotted dry and weighed to the nearest 0.01 mg using an electronic balance (AB135‐S, Mettler Toledo^®^, Zaventem, Belgium). At the end of the exposure period, three to five mosquito larvae per cup (depending on the survival) were randomly selected and weighed in the same way to obtain mean final wet mass per larva. For damselfly larvae, each larva was weighed at the start and end of the exposure period. Growth rates of both mosquito and damselfly larvae were calculated as (ln_final mass_−ln_initial mass_)/5 days (Dinh Van et al. 2013).

### Predation experiment

After the exposure experiment, mosquito larvae and damselfly larvae were jointly tested in the predation experiment. Mosquito larvae were used directly after their exposure period. Damselfly larvae were first starved for 24 h at their temperature‐by‐pesticide condition before being used in the predation trial to equalize hunger levels. For each predation trial, ten mosquito larvae and one damselfly larva of the same temperature‐by‐pesticide treatment combination were placed together in a 2.5‐L container (11 × 13 × 19 cm) filled with 1 L of their exposure medium and tested at their rearing temperature. Hence, both predators and prey were tested at the condition they experienced during the preceding exposure experiment. The number of replicates varied from 8 to 15 per damselfly latitude‐by‐temperature‐by‐pesticide treatment combination (total of 93 trials). Each mosquito larva and each damselfly larva were used in only one predation trial.

At the start of each 1‐h predation trial, the mosquito larvae were added 1 min before the introduction of the predator. Thereafter, we scored the position and activity of each mosquito larva every 10 min based on the protocol of Kesavaraju and Juliano (2010). Positions were classified into four categories: bottom, wall, water surface and water column. We also defined four activity categories (Kesavaraju and Juliano 2010): browsing (the mouthparts were in contact with the bottom or the wall of the container to graze for food), filtering (the larva was moving in the water column and made feeding movements with its mouthparts), thrashing (the larva was moving its body from side to side with vigorous flexion) and resting (the lava showed no movement). We calculated at each time point (*n* = 6) per container the percentage of mosquito larvae in each position and in each activity category and this throughout the predation trial (1 h).

During each predation trial, we also monitored the behavior of the damselfly larvae. Every 10 min we categorized the behavior as swimming, walking, head orientations toward the prey and inactivity (when the larva did not exhibit any of the other three categories) (see Janssens et al. 2014). At the end of the observation period, we calculated the frequency of each behavioral category per damselfly larva. Mass‐corrected predation rates by the damselfly larvae were calculated as the number of mosquito larvae eaten by a damselfly larva during 1 h divided by its body mass (see De Block et al. 2013).

### Statistical analyses

All statistical analyses were run in Statistica v.12 (StatSoft, Tulsa, OK, USA). To test for the effects of temperature, pesticide exposure and latitude of the damselflies on the response variables, we ran separate anovas. Survival data of both mosquitoes and damselflies during the exposure experiment were analyzed using logistic regression models with a binomial error structure. When analyzing effects on the damselfly larvae, we initially included population nested in latitude as a random factor; however, it had no effect on any of the response variables and we removed it from the final models.

For analyzing the detailed behaviors scored during the predation experiment, we first extracted principal components. Prior to the PCA, the mosquito behavioral data, which were expressed as percentages, were arcsine‐transformed while the damselfly behavioral data were log(x + 1)‐transformed. The resulting PC axes were then analyzed using anovas as mentioned above. When testing the effects of the temperature and pesticide treatments on mosquito behaviors, latitude of the damselfly predator was also included in the model; as it never had an effect, we removed it from the final models.

## Results

### Exposure experiment

**Figure 1:**
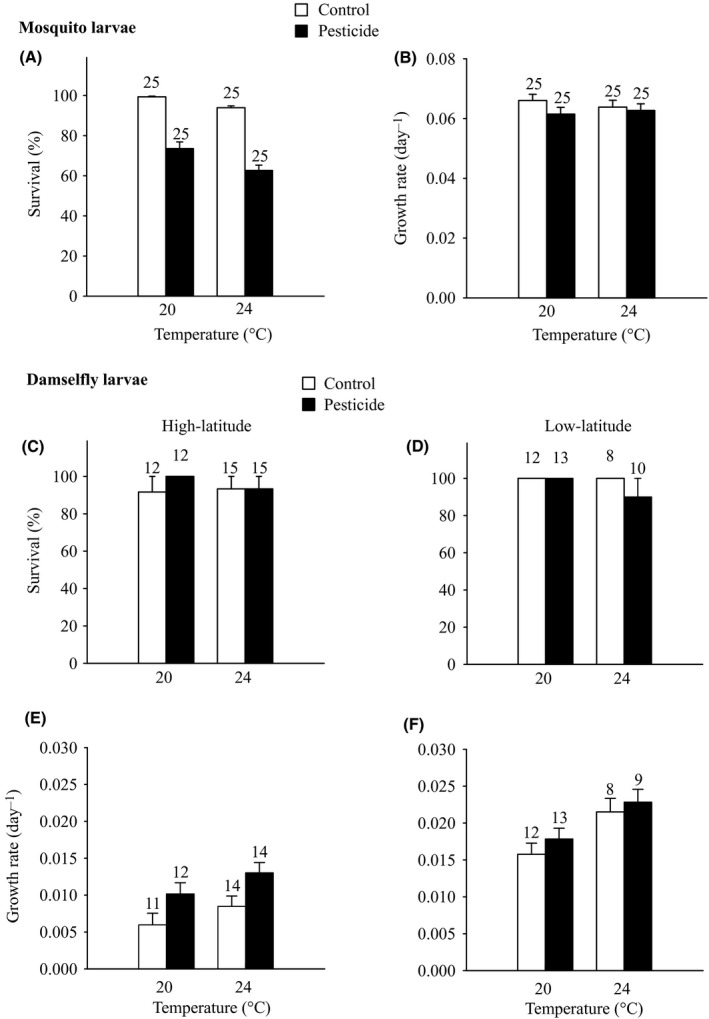
Survival (A, C, D) and growth rate (B, E, F) of *Culex pipiens* mosquito larvae (A, B) and *Ischnura elegans* damselfly larvae from low and high latitudes (C–F) as a function of the temperature and pesticide treatments. Given are least‐squares means with 1 SE. Numbers above bars indicate sample sizes.

Survival of mosquito larvae was ca. 100% at 20°C in the absence of the pesticide; survival was lower at the higher temperature and lower in the presence of the pesticide (Fig. 1A, Table 1). There was no interaction between the temperature and the pesticide treatments (Table 1). Growth rate was neither affected by the temperature nor by the pesticide treatment (Fig. 1B, Table 1).

**Table 1:** Results of anovas testing for the effects of temperature, pesticide exposure and latitude of origin of the damselfly larvae on survival and growth rate of *Culex pipiens* mosquito larvae and *Ischnura elegans* damselfly larvae during the exposure experiment

Effect	Mosquito larvae						Damselfly larvae					
	Survival			Growth rate			Survival			Growth rate		
	df	*χ*²	*P*	df1, df2	*F*	*P*	df	*χ*²	*P*	df1, df2	*F*	*P*
Temperature	1	16.09	**<0.001**	1, 96	0.05	0.819	1	0.86	0.352	1, 81	20.99	**<0.001**
Pesticide	1	170.43	**<0.001**	1, 96	1.66	0.200	1	0.01	0.913	1, 81	9.79	**0.0023**
Latitude							1	0.51	0.477	1, 81	124.42	**<0.001**
Temperature × Pesticide	1	3.70	0.055	1, 96	0.61	0.436	1	1.31	0.253	1, 81	0.01	0.922
Temperature × Latitude							1	0.10	0.746	1, 81	4.37	**0.040**
Pesticide × Latitude							1	1.31	0.235	1, 81	4.76	**0.032**
Temperature × Pesticide × Latitude							1	0.01	0.913	1, 81	0.15	0.701

Significant *P* values (*P* < 0.05) are indicated in bold.

Survival of the damselfly larvae was ca. 100% and not affected by the treatments (Fig. 1C,D, Table 1). Growth rate was higher in low‐latitude than in high‐latitude larvae (Fig. 1E,F, Table 1). The effects of both the temperature and the pesticide treatments differed between latitudes (Temperature × Latitude and Pesticide × Latitude, Fig. 1, Table 1). Follow‐up anovas indicated that growth rate was only higher at 24°C than at 20°C in low‐latitude larvae (*F*_1,37 _= 25.67, *P *<* *0.001), but not in high‐latitude larvae (*F*_1,44 _= 2.91, *P *=* *0.095). Growth rate only increased in larvae exposed to the pesticide compared to the control treatment in high‐latitude larvae (*F*_1,44 _= 13.22, *P < *0.001), but not in low‐latitude larvae (*F*_1,37 _= 0.52, *P *=* *0.476).

### Predation experiment

**Figure 2:**
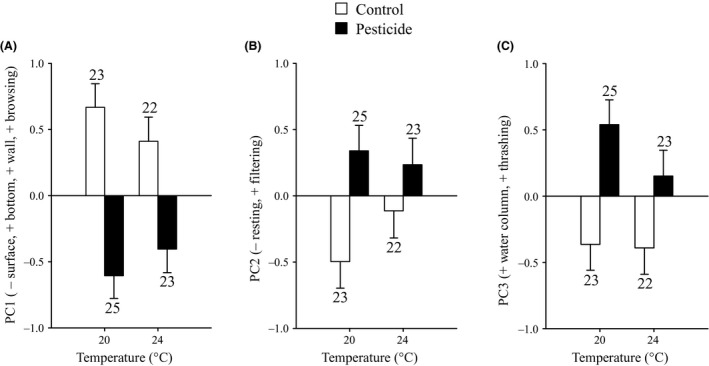
Behavioral PC scores (A: PC1; B: PC2; C: PC3) of *Culex pipiens* mosquito larvae during the predation experiment as a function of the temperature and pesticide treatments. Given are least‐squares means with 1 SE. Numbers above bars indicate sample sizes.

The PCA on the eight behavioral variables of the mosquito larvae resulted in three PC axes accounting for 80.1% of the total variation (Appendix S5). Mosquitoes with more positive scores on PC1 spent more time browsing on the bottom and at the walls of the container, and spent less time at the surface. Larvae with higher scores on PC2 spent more time filtering and less time resting. Larvae with higher values on PC3 spent more time thrashing in the water column. Exposure to the pesticide significantly affected each behavioral PC (Fig. 2, Table 2). Mosquito larvae exposed to the pesticide spent more time at the surface and browsed less frequently on the bottom, and at the walls (PC1), they showed more filtering and less time resting (PC2), and they spent more time thrashing in the water column (PC3).

**Table 2:** Results of anovas testing for the effects of temperature, pesticide exposure and latitude of origin of the damselfly larvae on the behavioral factor scores of *Culex pipiens* mosquito larvae (a), and *Ischnura elegans* damselfly larvae (b) during the predation experiment

Effect	PC1			PC2			PC3		
	df1, df2	*F*	*P*	df1, df2	*F*	*P*	df1, df2	*F*	*P*
(a) Mosquito larvae									
Temperature	1, 89	0.02	0.877	1, 89	0.49	0.487	1, 89	1.14	0.288
Pesticide	1, 89	34.62	**<0.001**	1, 89	8.85	**<0.004**	1, 89	13.87	**<0.001**
Temperature × Pesticide	1, 89	1.67	0.200	1, 89	1.50	0.224	1, 89	0.87	0.354
(b) Damselfly larvae									
Temperature	1, 85	1.21	0.275	1, 85	1.67	0.200	1, 85	1.33	0.252
Pesticide	1, 85	0.45	0.503	1, 85	1.70	0.196	1, 85	2.91	0.092
Latitude	1, 85	0.51	0.476	1, 85	0.00	0.952	1, 85	3.08	0.083
Temperature × Pesticide	1, 85	5.49	**0.021**	1, 85	1.74	0.191	1, 85	0.25	0.619
Temperature × Latitude	1, 85	0.55	0.460	1, 85	0.00	0.954	1, 85	0.19	0.663
Pesticide × Latitude	1, 85	0.16	0.692	1, 85	0.00	0.995	1, 85	4.88	**0.030**
Temperature × Pesticide × Latitude	1, 85	0.06	0.804	1, 85	0.00	0.965	1, 85	0.18	0.674

Significant *P* values (*P* < 0.05) are indicated in bold.

**Figure 3:**
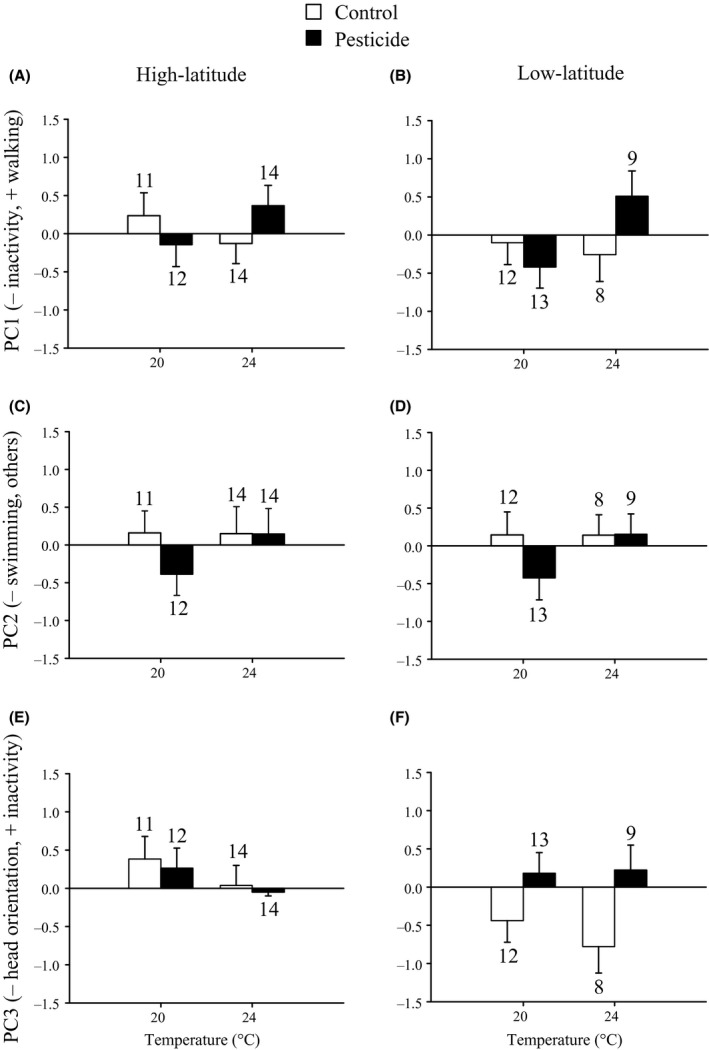
Behavioral PC scores of *Ischnura elegans* damselfly larvae from high (A, C, E) and low (B, D, F) latitudes during the predation experiment as a function of the temperature and pesticide treatments. Given are least‐squares means with 1 SE. Numbers above bars indicate sample sizes.

The PCA on the four behavioral variables of the damselfly larvae resulted in three PC axes that accounted for 97.8% of the total variation (Appendix S5). Larvae with more positive scores on PC1 spent more time walking and spent less time being inactive. Larvae with lower scores on PC2 spent more time swimming. Larvae with lower scores on PC3 showed more head orientations toward prey. The anovas showed that exposure to the pesticide affected behavioral PC1 and PC3 (Fig. 3, Table 2). Damselfly larvae exposed to the pesticide increased walking activity (PC1) but only at 24°C (Temperature × Pesticide). Pesticide‐exposed larvae spent more time being inactive and showed less head orientations (PC3) but only in low‐latitude damselfly larvae (Pesticide × Latitude).

**Figure 4:**
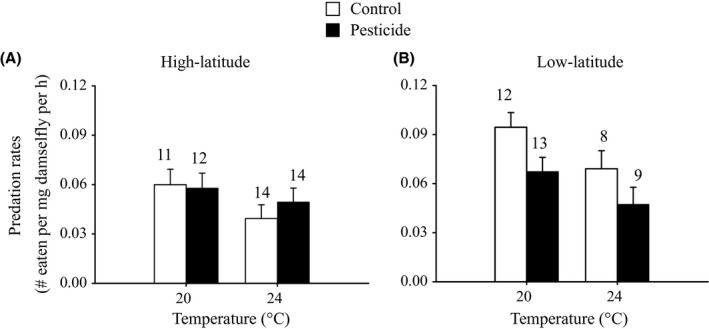
The number of *Culex pipiens* mosquito larvae eaten by *Ischnura elegans* damselfly larvae from high (A) and low (B) latitudes during the predation experiment as a function of the temperature and pesticide treatments. Given are least‐squares means with 1 SE. Numbers above bars indicate sample sizes.

Mass‐corrected predation rates by the damselfly larvae on the mosquito larvae were lower at 24°C than at 20°C (Fig. 4, Table 3). Low‐latitude damselfly larvae consumed more mosquito larvae than high‐latitude damselfly larvae, but only in the absence of the pesticide (Pesticide × Latitude, Fig. 4, Table 3). This Pesticide × Latitude interaction also indicated that exposure to the pesticide reduced predation rates but only in trials with low‐latitude damselfly larvae (Fig. 4).

**Table 3:** Results of anovas testing for the effects of temperature, pesticide exposure and latitude of origin of the damselfly larvae on the number of *Culex pipiens* mosquito larvae eaten in the predation experiment

Effect	Predation rate		
	df1, df2	*F*	*P*
Temperature	1, 85	7.87	**0.006**
Pesticide	1, 85	2.36	0.128
Latitude	1, 85	7.31	**0.008**
Temperature × Pesticide	1, 85	0.43	0.513
Temperature × Latitude	1, 85	0.38	0.537
Pesticide × Latitude	1, 85	4.59	**0.035**
Temperature × Pesticide × Latitude	1, 85	0.06	0.799

Significant *P* values (*P* < 0.05) are indicated in bold.

## Discussion

Our results indicate that both exposure to endosulfan and warming differentially affected life history and behavior of the mosquito prey and the damselfly predators, and shaped the outcome of their predator–prey interactions. Moreover, several of the treatment effects on the damselfly predators differed between high‐latitude and low‐latitude populations, likely driven by the evolution of faster growth rates (and associated higher vulnerability to the pesticide) and thermal adaptation in the low‐latitude populations. Key results were that endosulfan and warming only imposed mortality in the mosquito larvae, while endosulfan induced a growth rate increase in the high‐latitude damselfly larvae and temperature induced a growth rate increase in the low‐latitude damselfly larvae. Most importantly, predation rates on the mosquito larvae were reduced under warming and, in interactions with low‐latitude predators, also in the presence of the pesticide.

### Pesticide effects

The used endosulfan concentration differentially affected life history and behavior of the mosquito prey and the damselfly predators. Endosulfan imposed mortality on the mosquito larvae while the damselfly larvae instead only showed sublethal effects on growth rate. Specifically, exposure to the pesticide generated latitude‐specific effects consistent with the prediction that low‐latitude damselfly populations evolved a higher vulnerability to pesticides (see also Dinh Van et al. 2014a for the pesticide chlorpyrifos). In the presence of the pesticide, only high‐latitude larvae increased growth rate while only low‐latitude larvae reduced foraging activity (number of orientations toward prey). Given that high‐latitude larvae increased growth rate in the presence of the pesticide while their food intake did not change, the hormetic response was likely caused by a change in digestive efficiency. In line with this, endosulfan exposure caused an increase in growth rate in larvae of the damselfly *Coenagrion puella* which was not associated with an increased food intake but an increased efficiency of assimilating food (Campero et al. 2007). Given that hormetic responses are costly (Forbes 2000; McClure et al. 2014), we interpret this as only the less vulnerable populations, here the high‐latitude populations, being able to generate a hormetic growth response. This fits the general idea that adaptation to a warmer climate (here at the low latitude) will come at the cost of a reduced tolerance to contaminants (Moe et al. 2013). The higher vulnerability to pesticides in low‐latitude populations can be explained by their higher growth rates which through allocation trade‐offs likely result in less energy being allocated to defense (Sibly and Calow 1989; Congdon et al. 2001). Low‐latitude larvae of *I. elegans* evolved faster growth rates than high‐latitude larvae as they have multiple generations per year, hence have less time available per generation to complete a generation (Shama et al. 2011). In line with their higher energy demand, low‐latitude damselfly larvae consumed more mosquito larvae compared to high‐latitude larvae in the absence of the pesticide.

A key finding was that the evolution of different larval life histories and associated vulnerabilities to pesticides of the predators shaped predator–prey interactions in a latitude‐specific way. Specifically, the pesticide reduced predation rates on the mosquitoes but only in the low‐latitude damselfly larvae. Exposure to the pesticide had no main effect on the predation rates of damselfly larvae. Together with the observation that in the presence of the pesticide fewer mosquitoes were eaten, but only in interactions with low‐latitude damselflies, this indicates that it were primarily the pesticide effects on the predators that were driving the outcome of predator–prey interactions. This was supported by the observation that the pesticide reduced foraging activity (number of head orientations) of the predators but only in the low‐latitude populations. While many studies reported reduced predation rates in the presence of contaminants, very few tried to identify the underlying changes in the behaviors of predators and prey (reviewed in Fleeger et al. 2003; but see, e.g., Junges et al. 2012).

While the pesticide also affected all scored behaviors of the mosquito larvae, this apparently did not change their overall vulnerability to damselfly predators. Some of these behavioral changes (such as increased filtering and thrashing behaviors) likely made them easier to detect by the damselfly larvae. Yet, the pesticide‐induced changes in the position of the mosquito larvae (more at the surface and in the water column) likely reduced the encounter probability with the damselfly larvae and therefore may have counteracted the higher detection probability. This increased occurrence at the water surface may be a response to the increased oxygen need associated with an increased metabolic rate in the presence of the pesticide (Srivastava and Misra 1981). Note, however, that the latter mechanism together with the pesticide‐induced increase in thrashing behavior may make mosquito larvae more vulnerable to pelagic predators such as notonectids (Gimonneau et al. 2012).

Despite the mosquito prey suffering more from the pesticide than the damselfly predators in terms of survival, the pesticide, if anything, shaped the outcome of the predator–prey interactions in favor of the mosquito larvae. This seems counterintuitive and is in contrast with the prey stress model (Menge and Olson 1990) stating that when prey are more affected by the stressor than the predator, prey are expected to suffer higher predation rates in the presence of the stressor (for empirical support, see, e.g., Schulz and Dabrowski 2001). Yet, deviations from the prey stress model may not be unexpected (Junges et al. 2010). Indeed, in our study the pesticide‐induced mortality may have removed the mosquitoes with the slowest escape responses in the presence of the pesticide, so that the escape responses in the survivors that were used in the predation trials were no longer strongly affected by the pesticide.

### Temperature effects

Warming affected the mosquito prey and the damselfly predators in opposite ways and thereby shaped the outcome of predator–prey interactions. Mosquitoes suffered at the higher temperature as indicated by their higher mortality. This matches a previous study showing a higher mortality of *C. pipiens* at 24°C compared to 20°C (Ciota et al. 2014). In our study, this may reflect local thermal adaptation given that 20°C corresponds with the mean summer water temperatures of the mosquito source populations (Appendix S1). Instead, the damselfly larvae were not negatively affected by warming. Moreover, low‐latitude damselfly larvae were even growing faster at 24°C. This indicates a pattern of local thermal adaptation as previously observed for growth rate in this species (Shama et al. 2011; Dinh Van et al. 2014a).

Intriguingly, while only the prey suffered mortality at the high temperature, warming switched the outcome of predator–prey interactions in favor of the mosquitoes. This resembles the counterintuitive response pattern to the pesticide, yet here survival selection is a less likely explanation given that survival only slightly decreased under warming. The recorded behaviors of the mosquito prey and damselfly predators can also not explain the reduced predation rates under warming: temperature did not affect the mosquito behaviors, and there was no overall main effect of warming on the damselfly behaviors. Potentially, the mosquitoes became more efficient at evading predator attacks at the higher temperature because their escape speed increased more relative to the attack efficiency of the predators. Similarly, the stronger increase in escape speeds made mosquitofish less prone to predation by predatory bass under warming (Grigaltchik et al. 2012). In contrast to current findings, warming imposed higher predation rates of *I. elegans* on *Artemia* nauplii (Dinh Van et al. 2013, 2014a), and on *Daphnia magna* water fleas (De Block et al. 2013). Possibly, the latter two prey taxa do not increase escape speed to the same extent as mosquito larvae under warming hence cannot significantly lower the capture efficiency by the damselfly predators.

### Evolutionary perspectives with regard to global warming and mosquito control

How pest species will cope with pesticides and with their predators will be a major factor in shaping their control under global warming. Our results tentatively suggest that in the absence of evolution of the damselfly predators, a 4°C temperature increase as predicted by IPCC (2013) scenario RCP8.5 will change the outcome of predator–prey interactions at the high latitude in favor of the vector mosquitoes. In other words, all else remaining equal, biological control by damselfly predators would become less efficient. This is based on the general effect of decreased predation rates at 24°C. Note, however, that (assuming no thermal evolution of the mosquitoes) the higher temperature will also impose much higher direct mortality on the mosquitoes so that the changed predator–prey interactions likely will not translate into higher mosquito abundances. In case, high‐latitude populations of the damselfly predators, however, evolve toward the phenotype of low‐latitude populations currently living and adapted to 24°C, we may expect that the biological control of mosquitoes by damselfly larvae in the high latitudes will not change compared to the current situation. This is based on the observation that at 24°C the low‐latitude larvae had the same predation rates as the high‐latitude larvae currently living at 20°C. These predictions are, however, contingent on the limiting assumptions of the space‐for‐time substitution approach (Fukami and Wardle 2005; De Frenne et al. 2013; Elmendorf et al. 2015): (i) that besides temperature no other factors differ between latitudes that shape the studied traits (which is partly dealt with as we ran a common‐garden warming experiment), (ii) that populations will respond to changes in temperature over time in the same way that they will over space (Fukami and Wardle 2005) and (iii) that no interfering factors slow down the trait responses. While comparisons with other approaches proved space‐for‐time substitutions to be a valid approach (e.g., Elmendorf et al. 2015), the listed assumptions may limit the extent to which the results of our experiment can be used to simulate actual warming scenarios.

Another prediction based on our results is that latitude‐associated evolution may shape the outcome of predator–prey interactions under a scenario of invading low‐latitude predators. Poleward movements are very common and pronounced in damselflies (Hickling et al. 2006). Our results indicate that predation rates on mosquitoes at the high latitude will increase when they encounter invading southern damselflies. Yet, this is only true in the absence of the pesticide. In the presence of the pesticide, the evolved higher vulnerability to pesticides in the low‐latitude damselflies will result in equal predation rates compared to the high‐latitude damselflies. These latitude‐associated patterns are also directly relevant for current biological control of mosquitoes as they indicate that, all else being equal, predation rates by damselfly larvae will be higher at the low than at the high latitudes in the absence of pesticides.

Insights into how species interactions will change under global warming are outstanding applied evolutionary topics that are crucial to evaluate the potential of biological control in a warming world (Roderick et al. 2012). Specifically, we identified the potential role of evolution in shaping mosquito control by predators in a warming world, a largely overlooked aspect of how global warming may affect vector species and associated diseases (Kovats et al. 2001; Ramasamy and Surendran 2012; Parham et al. 2015). Our results indicate how the evolutionary differentiation of the damselfly predators between latitudes in life history and the associated differentiation in vulnerability to pesticides shape how a pesticide affects the current outcome of predator–prey interactions with a vector mosquito. Moreover, our results inform how *in situ* evolution and poleward movements of the predators may change these interactions at the high latitude under warming. Our results thereby illustrate the value of a space‐for‐time substitution approach (Fukami and Wardle 2005; De Frenne et al. 2013) to address applied evolutionary questions related to global warming.

## Data archiving statement

Data for this study are available from the Dryad Digital Repository: http://dx.doi.org/10.5061/dryad.15cn3.

## Supporting information


**Appendix S1.** Mean summer water temperature at the sites of origin of the mosquitoes.
**Table S1.** Mean summer water temperature (±1 SE) in the sites of origin of the mosquito culture in Germany.

**Appendix S2.** Mosquito culture.

**Appendix S3.** Characteristics of the study populations.
**Table S2.** Characteristics of the studied *Ischnura elegans* damselfly populations.
**Table S3.** Results of anovas testing for the effects of temperature, pesticide exposure, and population on survival and growth rate of *Ischnura elegan*s damselfly larvae during the exposure experiment for the set of three French populations.
**Table S4.** Results of anovas testing for the effects of temperature, pesticide exposure and population on the behavioral factor scores of *Ischnura elegans* damselfly larvae during the predation experiment for the set of three French populations.
**Table S5.** Results of anovas testing for the effects of temperature, pesticide exposure and population on predation rate of *Ischnura elegans* damselfly larvae in the predation experiment for the set of three French populations.

**Appendix S4.** Motivation endosulfan exposure concentration.

**Appendix S5.** Principal component analyses of the behavioral data.
**Table S6.** Principal component analyses of (a) the four positions and four activity scores of *Culex pipiens* mosquito larvae and (b) the four activity scores of *Ischnura elegans* damselfly larvae during the predation experiment. Percent variation explained by each PC is given within brackets. Factor loadings >0.5 or <−0.5 are indicated in bold.
