# Supplementary material for: Evolution determines how global warming and pesticide exposure will shape predator–prey interactions with vector mosquitoes
Source: Evol Appl. 2016 Jun 7;9(6):818–30. doi: 10.1111/eva.12390 (PMC4908467; doi:10.1111/eva.12390)
Supplement: Supplementary file 1 — Appendix S1. Mean summer water temperature at the sites of origin of the mosquitoes. Table S1. Mean summer water temperature (±1 SE) in the sites of origin of the mosquito culture in Germany. [file EVA-9-818-s001.docx]

**Appendix 1. Mean summer water temperature at the sites of origin of the mosquitoes**

To calculate the mean summer water temperatures in the three sites of origin of the mosquito culture, we first estimated realistic water temperatures for these sites by using the freshwater lake model Flake (Kirillin et al. 2011) following [Dinh Van et al. (2014](#_ENREF_11)). The Flake model must be parameterized by the mean depth, turbidity, wind fetch and the coordinates of the pond. As these data were not measured in the sites of origin we simulated ponds with an average depth of 1 m, turbidity of 0.1 m visibility and a standard wind fetch of 1 m, typical characteristics of habitats of *Culex pipiens* mosquitoes (shallow, eutrophic and stagnant ponds). We simulated the water temperature of the sites of origin for a period of 14 years from 1998 to 2012 during the summer months (from June to September). Based on these data we calculated the mean summer water temperature (Table S1).

**Table S1**. Mean summer water temperature (± 1SE) in the sites of origin of the mosquito culture in Germany

| Locality | Bammental | Berlin | Rotz |
| --- | --- | --- | --- |
| Coordinate | 49°34'N, 8°77'E | 52°51'N, 13°41'E | 49°40'N, 12°49'E |
| Mean temperature (°C) | 20.57 ± 0.11 | 19.75 ± 0.11 | 19.80 ± 0.11 |

**Literature cited**

Dinh Van, K., L. Janssens, S. Debecker, and R. Stoks. 2014. Temperature- and latitude-specific individual growth rates shape the vulnerability of damselfly larvae to a widespread pesticide. *Journal of Applied Ecolog*y **51**:919-928.

Kirillin, G., J. Hochschild, D. Mironov, A. Terzhevik, S. Golosov, and G. Nützmann. 2011. FLake-Global: Online lake model with worldwide coverage. *Environmental Modelling & Software* **26**:683-684.
