# Supplementary material for: Evolution determines how global warming and pesticide exposure will shape predator–prey interactions with vector mosquitoes
Source: Evol Appl. 2016 Jun 7;9(6):818–30. doi: 10.1111/eva.12390 (PMC4908467; doi:10.1111/eva.12390)
Supplement: Supplementary file 2 — Appendix S2. Mosquito culture. [file EVA-9-818-s002.docx]

**Appendix 2. Mosquito culture**

The mosquito culture was housed in a climate-controlled room at 20.3 ± 0.1°C, with a photoperiod of 14:10 h light:dark and a humidity of 60 ± 10 %. Egg clutches were incubated in plastic rearing trays (40 × 30 × 7.5 cm) filled with 5 L of aerated tap water. Three days after hatching, larvae were fed 0.1 mg per larva per day, five days a week with a mixture of Supradyn vitamin (3 %), Ovarit 7 cereal flakes (46 %) and wheat germs (51 %) (Op de Beeck et al. 2015). Larvae were kept at a density of ca. 200 individuals per rearing tray. After metamorphosis, adults were housed in small insectaries (30 × 30 × 30 cm) and supplied with a paper filter soaked in a 6 % glucose solution, which was replaced every other day. A petri dish filled with water and algae was provided for oviposition. We daily checked for freshly released egg clutches, which were incubated as described above.

During the 5-day exposure period, mosquito larvae were reared under the same conditions as during the pre-exposure period and daily fed 12.5 µL per larva of a 25 mg/mL solution of the same food mixture used in the pre-exposure period.

**Literature cited**

Op de Beeck, L., L. Janssens, and R. Stoks. 2015. Synthetic predator cues impair immune function and make the biological pesticide *Bti* more lethal for vector mosquitoes. *Ecological Applications*. <http://dx.doi.org/10.1890/15-0326.1>
