# Supplementary material for: Evolution determines how global warming and pesticide exposure will shape predator–prey interactions with vector mosquitoes
Source: Evol Appl. 2016 Jun 7;9(6):818–30. doi: 10.1111/eva.12390 (PMC4908467; doi:10.1111/eva.12390)
Supplement: Supplementary file 3 — Appendix S3. Characteristics of the study populations. Table S2. Characteristics of the studied Ischnura elegans damselfly populations. Table S3. Results of anovas testing for the effects of temperature, pesticide exposure, and population on survival and growth rate of Ischnura elegans damselfly larvae during the exposure experiment for the set of three French populations. Table S4. Results of anovas testing for the effects of temperature, pesticide exposure and population on the behavioral factor scores of Ischnura elegans damselfly larvae during the predation experiment for the set of three French populations. Table S5. Results of anovas testing for the effects of temperature, pesticide exposure and population on predation rate of Ischnura elegans damselfly larvae in the predation experiment for the set of three French populations. [file EVA-9-818-s003.docx]

**Appendix 3. Characteristics of the study populations**

Based on GIS analyses of the surrounding land use (distance to cropland, distance to woodland and woodland coverage), five of the study populations were not embedded by cropland and close to forest (Table S2) making it unlikely that they were affected by agriculture (Declerck et al. 2006). Only the French population Camaret-sur-Aigues was directly surrounded by cropland. We therefore tested in a set of separate ANOVAs whether this French population differed in its response to pesticide exposure compared to the other two French populations which were not embedded by cropland. These analyses revealed neither effects of population nor of interactions of population with the pesticide treatment (Tables S3-5). This indicates that the French population Camaret-sur-Aigues did not react differently than the two other French populations, suggesting it was not affected by pesticide exposure.

**Table S2.** Characteristics of the studied *Ischnura elegans* damselfly populations

| Damselfly population | Country | Coordinates | Distance to cropland (m) | Distance to woodland (m) | Woodland coverage (%) within a 200 m radius |
| --- | --- | --- | --- | --- | --- |
| St-Martin-de-Crau | France | 43°38'16.57"N, 4°50'49.06"E | 110 | 93 | 80 |
| Camaret-sur-Aigues | France | 44°9'1.47"N, 4°51'20.37"E | 0 | 3500 | 0 |
| Domaine de Valcros | France | 43°10'9.02"N, 6°16'11.36"E | 325 | 0 | 95 |
| Nöbbelövs mosse | Sweden | 55°44'5.98"N, 13°9'10.02"E | 247 | 70 | 40 |
| Erikso | Sweden | 58°56'4.90"N, 17°39'21.50"E | 120 | 80 | 70 |
| Ahl Hage | Denmark | 56°10'59.64"N, 10°39'1.69"E | 140 | 0 | 65 |

**Table S3.** Results of ANOVAs testing for the effects of temperature, pesticide exposure, and population on survival and growth rate of *Ischnura elegan*s damselfly larvae during the exposure experiment for the set of three French populations.

|  |  | Survival |  |  | Growth rate |  |
| --- | --- | --- | --- | --- | --- | --- |
|  | df | χ² | *P* | df1, df2 | F | *P* |
| Pesticide | 1 | 0.98 | 0.321 | 1, 29 | 0.22 | 0.646 |
| Population | 2 | 2.04 | 0.361 | 2, 29 | 1.92 | 0.164 |
| Pesticide × Population | 2 | 2.70 | 0.260 | 2, 29 | 1.33 | 0.280 |
| Temperature × Pesticide × Population | 2 | 3.12 | 0.210 | 2, 29 | 0.07 | 0.934 |

**Table S4.** Results of ANOVAs testing for the effects of temperature, pesticide exposure and population on the behavioral factor scores of *Ischnura elegans* damselfly larvae during the predation experiment for the set of three French populations.

| Effect |  | PC1 |  |  | PC2 |  |  | PC3 |  |
| --- | --- | --- | --- | --- | --- | --- | --- | --- | --- |
|  | df1, df2 | F | *P* | df1, df2 | F | *P* | df1, df2 | F | *P* |
| Temperature | 1, 29 | 1.04 | 0.317 | 1, 29 | 0.69 | 0.413 | 1, 29 | 0.31 | 0.583 |
| Pesticide | 1, 29 | 0.23 | 0.636 | 1, 29 | 0.73 | 0.401 | 1, 29 | 4.36 | **0.045** |
| Population | 2, 29 | 0.46 | 0.633 | 2, 29 | 0.67 | 0.520 | 2, 29 | 0.80 | 0.459 |
| Temperature × Pesticide | 1, 29 | 2.20 | 0.149 | 1, 29 | 0.73 | 0.400 | 1, 29 | 0.07 | 0.793 |
| Temperature × Population | 2, 29 | 0.11 | 0.892 | 2, 29 | 0.72 | 0.497 | 2, 29 | 2.91 | 0.070 |
| Pesticide × Population | 2, 29 | 0.04 | 0.962 | 2, 29 | 0.67 | 0.520 | 2, 29 | 0.37 | 0.694 |
| Temperature × Pesticide × Population | 2, 29 | 0.74 | 0.487 | 2, 29 | 0.69 | 0.508 | 2, 29 | 1.34 | 0.278 |

**Table S5.** Results of ANOVAs testing for the effects of temperature, pesticide exposure and population on predation rate of *Ischnura elegans* damselfly larvae in the predation experiment for the set of three French populations.

| Effect | Predation rate | | |
| --- | --- | --- | --- |
|  | df1, df2 | F | *P* |
| Temperature | 1, 29 | 2.93 | 0.097 |
| Pesticide | 1, 29 | 4.67 | **0.039** |
| Population | 2, 29 | 0.87 | 0.427 |
| Temperature × Pesticide | 1, 29 | 0.04 | 0.843 |
| Temperature × Population | 2, 29 | 0.21 | 0.815 |
| Pesticide × Population | 2, 29 | 0.58 | 0.565 |
| Temperature × Pesticide × Population | 2, 29 | 2.81 | 0.076 |

**Literature cited**

Declerck, S., T. De Bie, D. Ercken, H. Hampel, S. Schrijvers, J. Van Wichelen, V. Gillard, R. Mandiki, B. Losson, and D. Bauwens. 2006. Ecological characteristics of small farmland ponds: associations with land use practices at multiple spatial scales. *Biological conservation* 131:523-532.
