# Supplementary material for: Evolution determines how global warming and pesticide exposure will shape predator–prey interactions with vector mosquitoes
Source: Evol Appl. 2016 Jun 7;9(6):818–30. doi: 10.1111/eva.12390 (PMC4908467; doi:10.1111/eva.12390)
Supplement: Supplementary file 4 — Appendix S4. Motivation endosulfan exposure concentration. [file EVA-9-818-s004.docx]

**Appendix 4. Motivation endosulfan exposure concentration**

To determine the endosulfan concentration for the exposure experiment, we run a 5-day range finding experiment at 20°C with final instar larvae of both study species. We exposed animals to following concentrations: 0, 2, 4, 8, 16, 24, 28, 32 and 50 µg/L endosulfan. We renewed the medium after 48h and after 96h. These concentrations were chosen based on the LC_50_ _24h_ (66 µg/L) of this pesticide for *Culex pipiens* ([Mulla et al. 1964](#_ENREF_1)). The highest tested concentration was below the LC_50 24h_ (235 µg/L) of endosulfan for *Ischnura* damselfly larvae ([Schoettger and Glasgow 1970](#_ENREF_2)). The exposure experiment lasted for 5 days as the first mosquito larvae started pupating 6 days after they entered the final instar. In the range finding experiment, mosquitoes were exposed to the pesticide concentrations in 10 replicated groups of 20 larvae in 200 mL plastic cups filled with 125 mL of water control or pesticide medium. Damselfly larvae were exposed individually in the same type of cups (7 replicates per treatment). We scored mortality and growth rate across the exposure period.

We found no differences between the water control and the solvent control for mosquito larvae, this was true both for survival (water control: 97.73 ± 0.87 %, solvent control: 99.00 ± 0.91 %; ANOVA: F_1,19_ = 0.85, *P* = 0.367) and growth rate (water control: 0.068 ± 0.007 day^-1^, solvent control: 0.065 ± 0.007 day^-1^; ANOVA: F_1,19_ = 0.05, *P* = 0.826). No mosquito mortality was observed below 24 µg/L, and mortality gradually increased at higher concentrations (24 µg/L: 18 %, 28 µg/L: 35 %, 32 µg/L: 47 %, 50 µg/L: 94 %). No growth effects on mosquitoes were observed (ANOVA: F_8,74_ = 1.25, *P* = 0.29). For damselfly larvae, no mortality occurred within this concentration range, yet compared to the control (mean ± 1 SE, control: 0.010 ± 0.001 day^-1^) growth rate was considerably reduced at 28 µg/L (0.006 ± 0.001 day^-1^) but not at 24 µg/L (0.009 ± 0.001 day^-1^) (ANOVA: F_2,18_ = 4.13, *P* = 0.033; Tukey post hoc tests comparing with the water control, at 28 µg/L: *P* = 0.039, at 24 µg/L: *P* = 0.900). Note that the absence of a difference in survival and growth rate between the water control and 24 µg/L endosulfan also indicates that the used aceton concentration (1 µl/ml) did not affect the damselfly larvae. Therefore, we selected an endosulfan concentration of 28 µg/L for the exposure experiment. For the control we used a water control instead of a solvent control.

**Literature cited**

Mulla, M. S., R. L. Metcalf, and G. Kats. 1964. Evaluation of new mosquito larvicides, with notes on resistant strains. *Mosquito News* **24**:312-319.

Schoettger, R. A., and L. L. Glasgow. 1970. Toxicology of thiodan in several fish and aquatic invertebrates. *Investigation in Fish Control* Series Report 35. US Department of Interior Fish and Wildlife, Washington, D.C.
