# Supplementary material for: Evolution determines how global warming and pesticide exposure will shape predator–prey interactions with vector mosquitoes
Source: Evol Appl. 2016 Jun 7;9(6):818–30. doi: 10.1111/eva.12390 (PMC4908467; doi:10.1111/eva.12390)
Supplement: Supplementary file 5 — Appendix S5. Principal component analyses of the behavioral data. Table S6. Principal component analyses of (a) the four positions and four activity scores of Culex pipiens mosquito larvae and (b) the four activity scores of Ischnura elegans damselfly larvae during the predation experiment. Percent variation explained by each PC is given within brackets. Factor loadings >0.5 or <−0.5 are indicated in bold. [file EVA-9-818-s005.docx]

**Appendix 5.** **Principal component analyses of the behavioral data**

**Table S6.** Principal component analyses of (a) the four positions and four activity scores of Culex pipiens mosquito larvae and (b) the four activity scores of *Ischnura elegans* damselfly larvae during the predation experiment. Percent variation explained by each PC is given within brackets. Factor loadings >0.5 or < -0.5 are indicated in bold.

| (a) Mosquito larvae |  |  |  |
| --- | --- | --- | --- |
| Variable | PC1 (40.3%) | PC2 (27.0%) | PC3 (13.1%) |
| Bottom | **0.86** | 0.06 | -0.22 |
| Wall | **0.74** | -0.24 | 0.11 |
| Water column | 0.12 | 0.08 | **0.90** |
| Surface | **-0.95** | -0.05 | -0.03 |
| Browsing | **0.88** | 0.26 | -0.09 |
| Filtering | -0.08 | **0.94** | 0.10 |
| Thrashing | -0.38 | 0.25 | **0.63** |
| Resting | -0.18 | **-0.91** | -0.19 |
| 1. Damselfly larvae | |  |  |
| Variable | \| PC1 (49.9%) \| \| --- \| | \| PC2 (25.1%) \| \| --- \| | \| PC3 (22.8%) \| \| --- \| |
| Head orientation | 0.10 | -0.00 | **-0.99** |
| Swimming | 0.01 | **-1.00** | -0.01 |
| Walking | **0.99** | 0.03 | 0.03 |
| Inactive | **-0.82** | 0.07 | **0.52** |
